# 

**Published:** 1893-12

**Authors:** 


### CONTENTS:

ORIGINAL ARTICLES: PAGE

Hydrated Chloral—Chloral Hydrate. Chloral. By John R. Anderson, D.V.S...... 365
Glanders-Farcy and Mallein. By Simon J. J. Harger, Professor of Anatomy and Zootechnics, University of Pennsylvania...... 370
Cuterebra Emasculator in Dog. By Cecil French...... 379
Report of the Tuberculosis Committee. By A. W. Clement, V.S...... 380
Millet Disease.—Concluded from page 322. By T. D. Hinebauch, M.S., V.S...... 396
Osteo-porosis. By Dr. Claude D. Morris...... 397

EDITORIAL:

Veterinary Titles...... 411
New York State Veterinary Medical Society Notice...... 413

SOCIETY PROCEEDINGS:

Massachusetts Veterinary Association...... 414
Keystone Veterinary Medical Association...... 419
Society for the Study of Comparative Psychology in Connection with the Faculty of Comparative Medicine and Veterinary Science of McGill University, Montreal, Canada...... 420
Chicago Veterinary College.—A New Veterinary Degree...... 425
New York State Veterinary Medical Society.—Concluded from page 361...... 426

VETERINARY GRADUATES, 1893:

New York College Veterinary Surgeons...... 431
Chicago Veterinary College...... 432
American Veterinary College...... 434
McGill University, Montreal...... 435
Iowa Agricultural College...... 435
Ontario Veterinary College...... 436
Ohio Veterinary College...... 439
University of Pennsylvania...... 440
Harvard University...... 440

RECORD OF FASTEST TIME MADE BY HORSES...... 441

